# "Single-Surgeon" versus "Dual-Surgeon" Robot-Assisted Radical Prostatectomy and Pelvic Lymph-nodes Dissection: Comparative Analysis of Perioperative Outcomes

**DOI:** 10.1590/S1677-5538.IBJU.2023.0348

**Published:** 2024-02-07

**Authors:** Riccardo Bertolo, Marco Carilli, Michele Antonucci, Francesco Maiorino, Pierluigi Bove, Matteo Vittori

**Affiliations:** 1 San Carlo di Nancy Hospital Department of Urology Rome Italy Department of Urology, San Carlo di Nancy Hospital, Rome, Italy;; 2 Tor Vergata University of Rome Department of Surgery Urology Unit Rome Italy Urology Unit, Department of Surgery, Tor Vergata University of Rome, Rome, Italy

**Keywords:** Prostatic Neoplasms, Prostatectomy, Minimally Invasive Surgical Procedures

## Abstract

**Purpose:**

To compare the perioperative outcomes of robot-assisted radical prostatectomy (RARP) with pelvic lymph-nodes dissection (PLND) when the same surgeon performs RARP and PLND versus one surgeon performs RARP and another surgeon performs PLND.

**Materials and Methods:**

From January 2022 to March 2023, data of consecutive patients who underwent RARP with PLND were prospectively collected. The surgeries were performed by two "young" surgeons with detailed profile. Specifically for the study purpose, one surgeon performed RARP, and the other surgeon performed PLND. A set of surgeries performed according to the standard setup (i.e., the same surgeon performing both RARP and PLND) was retrieved from the institutional database and used as comparator arm. To test the study hypothesis, patients were divided into two groups: "dual-surgeon" versus "single-surgeon".

**Results:**

Fifty patients underwent RARP and PLND performed according to dual-surgeon setup and were compared to the last 50 procedures performed according to the standard single-surgeon setup. Patients in the groups had comparable baseline characteristics. Dual-surgeon interventions had significantly shorter median total operative (194 [IQR 178–215] versus 174 [IQR 146–195] minutes, p<0.001) and console time (173 [IQR 158–194] versus 154 [IQR 129–170] minutes, p<0.001). No significant differences were found in terms of blood loss, intraoperative complications, postoperative outcomes, and final pathology results.

**Conclusions:**

The present analysis found that when RARP and PLND are split onto two surgeons, the operative time is shorter by 20 minutes compared to when a single surgeon performs RARP and PLND. This is an interesting finding that could sponsor further studies.

## INTRODUCTION

Robot-assisted radical prostatectomy (RARP) is nowadays an established minimally invasive option for the treatment of prostate cancer (1). Patients who underwent RARP do benefit from the inherent advantages of laparoscopic surgery paired to superior functional and oncological outcomes (2).

During RARP, the surgeon stays focused on the console, looking at the three-dimensional surgical field. The possibility of adjusting the console to the surgeon's individual preferences may increase the comfort of her / his workstation. On the other hand, working for hours at a monitor-based workstation has been reported to be exhausting (3-7)

Despite the understanding of this phenomenon, there is scarce literature evaluating the surgical outcomes of either prolonged or consecutive surgical procedures.

In daily practice our surgeons noticed the potential for showing a different performance during RARP, if RARP is performed with or without pelvic lymph-nodes dissection (PLND).

To test such hypothesis, we started with splitting the two steps of the procedure (RARP and PLND) among two surgeons. The aim of the study was to compare the perioperative outcomes of RARP with PLND when the same surgeon performs both RARP and PLND versus one surgeon performs RARP and another surgeon performs PLND.

## MATERIALS AND METHODS

### Patients

From January 2022 to March 2023, perioperative data of consecutive patients who underwent both RARP and PLND were prospectively collected into an institutional dedicated database (IRB 845/CE Lazio 1). PLND was planned based upon the individual patient's risk of harbouring positive lymph-nodes, as estimated by the validated Gandaglia's nomogram (8).

The surgeries were performed by two surgeons with detailed profile (< 40 years-old, > 1000 robotic surgeries assisted at bedside, > 200 robotic surgical interventions performed as console surgeon of which > 150 RARP interventions).

For the purpose of the study, within the "experimental" arm, one surgeon performed RARP and the other one performed PLND (and vice versa for each consecutive case).

A set of surgeries performed according to the standard setup at our institution (i.e., the same surgeon performs both RARP and PLND) was retrieved from the institutional database and used as comparator arm. The patients were divided into two groups: Group "dual-surgeon" versus Group "single-surgeon".

### Surgical technique

All patients underwent Da Vinci Xi extended PLND and RARP with transperitoneal approach. Briefly, extended PLND was performed as previously described, as including the removal of the nodes overlying the external iliac artery and vein, the nodes within the obturator fossa located cranially and caudally to the obturator nerve, and the nodes medial and lateral to the internal iliac artery (9).

For RARP, an incision of the parietal peritoneum (without transecting the urachus) was made to access the retropubic space. The prostate was identified and dissected free from the periprostatic fat. The endopelvic fascia was incised on both sides, while preserving the pubo-prostatic ligaments. The bladder neck was dissected according to a "bladder neck sparing" technique, whenever feasible. To gain access to the retro-trigonal space, the muscular fibres that anchor the bladder to the base of the prostate were dissected while leaving a lingula of muscular tissue attached to the posterior aspect of the bladder neck (the so-called "retro-trigonal fascia"). The anterior layer of the Denonvillers' fascia was incised, then the seminal vesicles were identified and dissected. The posterior layer of the Denonvilliers' fascia was incised in an "inverse U-shape" in the proximity of the prostate gland, to gain access to the perirectal space that was developed as much as possible towards the posterior prostate apex.

The visceral layer of the endopelvic fascia and the underlying apron still covering the anterior surface of the prostate were then incised while sparing the pubo-prostatic ligaments. The prostate apex was managed via a combination of blunt and sharp dissection, until the urethra was incised. The deep venous complex was selectively closed by 3/0 "barbed" suture. The posterior reconstruction was performed in a double layer by using a 3/0 "barbed" suture. To create the first layer, the needle was passed into the median raphe and the cranial portion of the previously divided retro-trigonal fascia. The second layer involved the bladder neck and the posterior aspect of the urethra. The urethro-vesical anastomosis was performed by using two 3/0 "barbed" hemi-running sutures, according to a modified Van Velthoven's single-knot technique (10).

### Outcome measurements

Demographic characteristics as well as intra- and post-operative data of patients in both groups were compared. Preoperative patient's and disease's information included age, body mass index, Charlson's comorbidity index, and the International Society of Urological Pathology (ISUP) prognostic grade groups at prostate biopsy (11).

Perioperative outcomes included operative time, estimated blood loss, intraoperative complications, length of hospital stay, catheterization time, and complications, as classified according to the Clavien-Dindo classification (12).

Surgical specimens underwent final pathology examination to have grading, staging (both for prostate and retrieved lymph-nodes), surgical margin status, and prostate size reported.

### Statistical Analysis

Descriptive statistics for the Single-surgeon and the Dual-surgeon groups were summarized by median and interquartile range (IQR) for continuous variables. Frequencies and proportions were used for categorical variables, as appropriate.

Continuous variables were analysed with the Wilcoxon's test. Differences between categorical variables were assessed by using the chi-square and Fisher's exact tests, as appropriate. Multivariate analysis was performed to account for independent factors impacting on the observed differences between the groups.

The minimal required sample size was 48 versus 48 patients, calculated by hypothesizing an expected difference of 20 minutes between the arms at the time of study design (alpha = 0.05, beta = 0.2, power = 0.8).

Statistical analyses were performed by using SPSS software v.24.0 (IBM Corp, Armonk, NY, USA). All tests were two sided, with significance set at p-value < 0.05.

## RESULTS

Fifty patients underwent RARP and PLND performed according to a dual-surgeon setup and were compared to the last 50 procedures retrieved from the institutional dataset, performed according to the standard single-surgeon setup.

The patients in the groups had comparable demographic and disease characteristics (Table-1).

**Table 1:** Distribution of baseline characteristics.

		Single-Surgeon (n = 50)	Dual-Surgeon (n = 50)	p-value
Age, median (IQR), years		67 (62 – 72)	67 (59 – 71)	0.9
PSA, median, (IQR), ng/mL		8.5 (5.9 – 12.7)	8.6 (6.5 – 12.0)	0.7
BMI, median (IQR), kg/m2		25.3 (24.2 – 28.1)	25.4 (23.4 – 28.4)	0.9
**Charlson comorbidity index, no. (%)**				0.8
	0-1	19 (38)	17 (34)	
	2-3	29 (58)	30 (60)	
	≥ 4	2 (4)	3 (6)	
**Biopsy ISUP grade group, no. (%)**				0.9
	Grade group 1	2 (4)	2 (4)	
	Grade group 2	14 (28)	13 (26)	
	Grade group 3	19 (38)	18 (36)	
	Grade group 4	12 (24)	14 (28)	
	Grade group 5	3 (6)	3 (6)	

Interventions performed according to the dual-surgeon setup had significantly shorter median total operative (194 [IQR 178 – 215] versus 174 [IQR 146 – 195] minutes, p < 0.001) and console time (173 [IQR 158 – 194] versus 154 [IQR 129 – 170] minutes, p < 0.001).

No significant differences were found in terms of estimated blood loss and occurrence of intraoperative complications. No significant differences were found in terms of postoperative outcomes of interest, including final pathology results (Table-2). Multivariate analysis did not find any independent factors impacting on the observed differences between the groups in operative and console time.

**Table 2:** Distribution of Perioperative Outcomes and Pathology Outcomes.

		Single-Surgeon (n = 50)	Dual-Surgeon (n = 50)	p-value
**Perioperative Outcomes**				
Total operative time, median (IQR), min		194 (178-215)	174 (146-195)	< 0.001
Console time, median (IQR), min		173 (158-194)	154 (129-170)	< 0.001
Estimated blood loss, median (IQR), mL		150 (100-200)	150 (100-250)	0.2
Nerve Sparing (at least unilateral), no. (%)		8 (16)	9 (18)	0.8
Intraoperative complications, no. (%)		2 (4)	1 (2)	0.6
Catheterization time, median (IQR), days		5 (4-6)	5 (4-6)	--
Grade ≥ 2 postoperative complications, no (%)		4 (8)	3 (6)	0.7
Postoperative readmissions, no (%)		1 (2)	1 (2)	--
**Pathology Outcomes**				
Prostate weight, median (IQR), g		56 (40 – 62)	53 (43 – 67)	0.5
Pathological ISUP grade group, no. (%)				0.6
	Grade group 1	--	--	
	Grade group 2	14 (28)	11 (22)	
	Grade group 3	19 (38)	19 (38)	
	Grade group 4	9 (18)	10 (20)	
	Grade group 5	8 (16)	10 (20)	
Pathological T stage, no. (%)				0.8
	pT2	21 (42)	20 (40)	
	≥ pT3	29 (58)	30 (60)	
Pathological N stage, no. (%)				0.8
	pN0	41 (82)	42 (84)	
	pN+	9 (18)	8 (16)	
No. lymph-nodes removed, median (IQR)		14 (11 – 20)	14 (12 – 19)	0.8
Positive surgical margins, no (%)				0.8
	Total	12 (24)	11 (22)	
	pT2 cases	5	5	
	pT3 cases	7	6	

## DISCUSSION

The fact that the surgeon has to stay focused on a monitor-based workstation (i.e. the robotic console) during RARP has reported to be exhausting [2-6]. Martinschek et al. assessed human concentration via a computer-assisted attention test at the time that surgeons were performing RARP. They found an association between prolonged console time and decreased concentration. Furthermore, when consecutive surgeries were performed, they reported an increase in the total number of errors (13). In contrast, Bukavina et al. recently analysed a multi-institutional retrospective cohort aimed to determine whether the outcomes of RARP differed when two RARP interventions were performed one after another by the same team. In their hands, case sequence was found not associated with operative time, positive margins, and lymph-nodes yield (14). Similarly, Bagrodia et al. reported that performance of several consecutive complex urological procedures (including RARP) was not associated with the worsening of outcomes (15).

Despite some understanding, literature lacks in evaluating the outcomes of "prolonged" robotic surgeries. In this setting, we felt our research of interest. The most "pathos" step of surgery for prostate cancer is PLND, which is typically performed as first act. At the end of such a "procedure in the procedure", the surgeon can experience a sort of "fall" in the adrenergic tone. We hypothesized that this could be able to impact on the performance of the RARP performed just after.

The present analysis of our institutional data found that when RARP and PLND are split onto two surgeons, the operative time is shorter by 20 minutes if compared to when a single surgeon performs both RARP and PLND.

The reader could argue whether such a reduction in operative time was relevant. It has been demonstrated that having an experienced team able to anticipate the surgeon's actions can reduce the operative time: this does increase the efficiency in the operative room dedicated to robotic surgery (16). However, it is challenging to evaluate the reduction in the absolute costs of RARP relative to such an increased "efficiency".

Lone et al. tried to mitigate the financial burden of RARP and prospectively implemented a cost reduction plan that included a lower number of robotic instruments used per surgery. The authors investigated whether these changes did impact on the costs of RARP as well as the perioperative outcomes. Interestingly, the authors found that their cost reduction plan corresponded to a significant decrease in the console time and calculated $200 savings per case (95% [CI] $150-$250, p<0.0001) per each 15-minute reductions in surgery time (17).

In addition to the interest in saving "out-of-pocket" costs, the shorter surgical times (particularly if the reader adds up the time saved after 2-3 procedures/day) could translate into the potential for performing an additional procedure within the same 12-hour day shift. This, in turn, could translate into a higher volume of surgeries with a 1-year time horizon and increase the amortization of the robotic platform costs from the perspective of the healthcare system (18). Moreover, we are unable to value the invaluable fact that, with the configuration suggested herein, the exposure to robotic surgery is doubled up for each surgeon.

Finally, longer operative time during RARP is reported to be associated with longer hospitalization, longer catheterization, and higher likelihood of adverse events. This is why we believe that anything that can be done for reducing the operating time should be welcome (19).

One issue that could detract from the value of our results could be the experience of the surgeons involved. Specifically, the reader could argue that the latter set of procedures was performed by surgeons with wider experience (they had 50 more surgeries on their shoulders). We prove that this is not the case. A recent systematic review from the United Kingdom focused on the learning curves of major laparoscopic and robotic procedures in urology reported a considerable variation in the definitions of outcome measures and performance thresholds of RARP. Specifically, the learning curve for operative time was identified as ranging from 10 to 250 cases for RARP (20). Ambinder et al. performed a retrospective analysis of RARP procedures performed by a new attending surgeon and observed a 25-case learning curve for a fellowship-trained urologist to achieve stable operative performance in RARP. Procedural components demonstrated variable learning curves including the urethro-vesical anastomosis that required upward of 50 cases (21).

A panel of experts developed and validated a modular training and assessment pathway via Healthcare Failure Mode and Effect Analysis for trainees undertaking RARP and evaluated learning curves for procedural steps. The authors demonstrated plateaus for many steps of RARP, including anterior and posterior bladder neck dissection (16 and 18 cases, respectively), posterior dissection (9 cases), dissection of prostatic pedicles and seminal vesicles (15 cases), and anastomosis (17 cases) (22). If one does not like to base upon such arbitrary cut-offs of the total cases, Tamhankar et al. analysed a large dataset of RARP interventions and demonstrated that it takes about 300 cases and nearly 4 years to standardise operative and console times, with a requirement of around 80 cases per year for a single surgical team to optimise the outcomes of RARP (23).

Finally, it has been reported that the quality of PLND during RARP seems to be not related to the number of procedures performed, allowing for the removal of a number of lymph-nodes by a less experienced surgeon that is clinically comparable to that of a high-volume surgeon (24).

In summary, the experience matured by the Centre and the surgeons involved within the setting of the present study was by far enough to meaningfully discuss the observed difference in operative time.

We acknowledge the limitations of the study. First, although the data of the experimental cohort were prospectively collected, our analysis was retrospective and partly based on a historical cohort, with inherent bias. Moreover, the study considered subjective inclusion criteria. We might have missed the full establishment of causal inference, due to potential confounders that remained not observed. These, alone, could have contributed to the observed difference in operative time. Second, we did not control for the bedside assistant experience, although all the bedside assistants involved had comparable background. On the other hand, we underline that our results could be not fully generalized, given the surgical profile of the involved console surgeons. As reported above, they had assisted > 1000 robotic procedures before the study start. To note, it has been demonstrated that serving as a bedside surgeon before performing RARP improves the outcomes of the surgeon once at the console (25).

Third, being the analysis focused on early outcomes of RARP, biochemical recurrence was not reported with only surgical margins status provided as a proxy. Interestingly, within a multicentric experience, Bravi et al. investigated the relationship between biochemical recurrence after RARP and surgeon's experience. As opposed to open and laparoscopic radical prostatectomy series, the authors found that "novice" surgeons performing RARP achieved adequate cancer control since the early phase of their career. In this setting, we believe that further research should explore why the learning curve for RARP differs from prior findings about open and laparoscopic surgery. It has been hypothesized that surgical education (including simulation) and the adoption of performance metrics are contributing at flattening the learning curve of the new generations of surgeons (26).

Finally, although of paramount importance in the quality assessment of RARP, continence and potency outcomes were not reported being beyond the scopes of our study (27).

Notwithstanding the aforementioned limitations, our analysis suggests an interesting impact from splitting RARP and PLND onto two surgeons, showing a certain shortening in the operative time which we feel worth of further investigation.

## CONCLUSIONS

The present analysis found that when RARP and PLND are split onto two surgeons, the operative time is shorter by 20 minutes compared to when a single surgeon performs RARP and PLND. This is an interesting finding that could sponsor further studies.
